# Mutation Analysis of the *APC* Gene in a Chinese FAP Pedigree with Unusual Phenotype

**DOI:** 10.5402/2011/909121

**Published:** 2011-03-15

**Authors:** S. Chen, J. Zhou, X. Zhang, X. Zhou, M. Zhu, Y. Zhang, G. Ma, J. Li

**Affiliations:** ^1^Laboratory of Genetics and Molecular Biology, Jiangsu Institute of Cancer Research, Nanjing 210009, China; ^2^Department of General Surgery, Jiangsu Cancer Hospital, Nanjing 210009, China

## Abstract

*Background and Aim*. Germline mutations of the adenomatous polyposis coli (*APC*) gene cause familial adenomatous polyposis (FAP), an autosomal dominant inherited disease mainly characterized by colorectal adenomatous polyposis. Genetic studies of FAP have shown that somatic APC mutations are dependent on the position of the germline APC mutation. However, the molecular mechanism underlying these genotype-phenotype associations for APC in Chinese remain largely unknown. *Patients and Methods*. In this study, we investigated the *APC* gene mutation in a Chinese FAP family by systematic screening with multiplex ligation-dependent probe amplification (MLPA), denaturing high-performance liquid chromatography (dHPLC), and DNA sequencing. Promoter methylation was detected by methylation-specific PCR. *Results*. The identical germline mutation c.1999 C>T (Q667X) of *APC* was identified in 5 affected members, among which 2 members carried somatic mutations of *APC*, one with promoter hypermethylation and the other with loss of wild-type allele in their adenomas. The somatic mutations were shown connected with the disease severity, demonstrating a unique genotype-phenotype association in this FAP pedigree. *Conclusion*. The study revealed the existence of novel pathogenic mutations in Chinese patients with FAP. Somatic mutations are of particular interest because of the unusual phenotypic features shown by patients.

## 1. Introduction

Familial adenomatous polyposis (FAP; MIM #175100) is a common hereditary syndrome characterized by early onset form of colorectal cancer based on multiple adenomas in the colon and rectum [1]. The penetrance of the genetic defect is approximately 100%. The responsible gene for FAP, the adenomatous polyposis coli (*APC*) gene, was isolated and localized to chromosome 5q21-22 by positional cloning [2, 3]. More than 900 different mutations have been registered in the Human Gene Mutation Database (http://www.hgmd.cf.ac.uk/ac/). Most mutations in APC are nonsense or frameshift mutations that cause premature truncation of the APC protein, and more than 60% have been localized within exon 15 [4, 5]. In addition, several studies revealed that the exon-spanning fragment variations in *APC* gene may be a frequent cause of FAP [6, 7]. In several studies, an association between the location of the APC mutation and the phenotype in FAP patients has been described [8]. Identification of the APC mutation in one affected individual of a family opens the possibility of testing the at-risk members for the same mutation, thus preventing the development of colorectal cancer by the prophylactic removal of the bowel. Although the *APC* gene has been extensively studied in the Caucasian population, it has not been widely described in the Chinese population. The molecular mechanisms underlying the genotype-phenotype associations for *APC* remain largely unknown [9, 10]. Furthermore, the possible role of APC inactivation by methylation need to be further assessed. In this study, we screened the whole coding region of *APC* gene by MLPA, dHPLC, and DNA sequencing to identify the germline and somatic mutations in all of the affected members of a suspicious FAP family and discussed the genotype-phenotype association.

## 2. Patients and Methods

**Figure 1:**
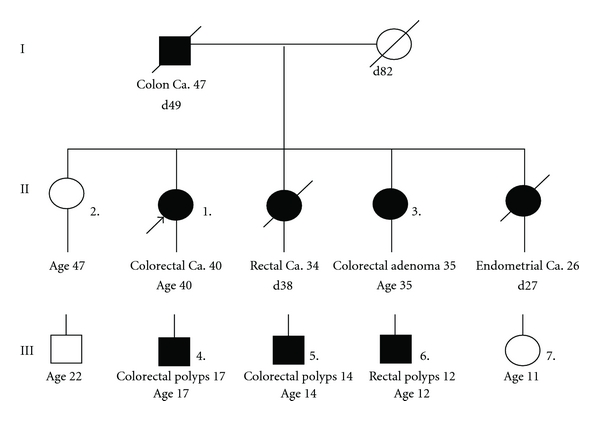
Pedigree of the family with autosomally dominant inheritance of familial adenomatous coli. d = died; Age = current age; Blackened symbols indicate affected individuals. Proband is denoted with a filled arrowhead, and roman numbers indicate generations.

The FAP family pedigree (Figure 1) consists of 7 members, with 5 affected and 2 unaffected members. The proband was a 40-year-old female presenting to our hospital in August, 2005, with increasing intermittent rectal bleeding and abdominal pain for several months. Her family history included her father and two younger sisters, who died of rectal cancer in the age of 49, colorectal cancer at age of 38, and endometrial cancer at age of 27, respectively. Colonoscopy performed in the proband revealed numerous polyps carpeting the entire colon and rectum. Histological examination of biopsied specimens revealed findings of poorly differentiated adenocarcinomas, and the other polyps were diagnosed as tubular adenomas with mainly heavy-grade dysplasia. On the basis of these findings, the patient was diagnosed to have FAP. With informed consent from the patient and her family members, we collected their peripheral blood. For the numbered family members, colonoscopy examination was performed, and the found adenomas, adenocarcinomas, and the paired normal tissues were obtained. Each biopsy was assessed by the analysis of hematoxylin and eosin-stained sections. Fifteen control subjects were selected from medical checkup people in our hospital free from cancer.

DNA was isolated from peripheral blood and obtained tissue from consenting family members with the Qiamp DNA Blood and Tissue Extraction kit (Qiagen Corp, Germany), respectively. MLPA analysis to determine the large fragment duplication or deletion and allelic loss in *APC* gene was performed using the Salsa MLPA kit P043 APC (MRC-Holland Corp, Amsterdam, The Netherlands) according to the protocol supplied with the kit with a few modifications according to the condition in our laboratory. The WAVE System 3500 (Transgenomic, Inc., Omaha, NE) and associated WAVE-Maker 4.1 software were used to analyze the exons 1 to 15 of *APC* gene, each of them in the range of 200–500 bp. Preparation and analysis of the PCR products was carried out as previously described [11]. In brief, oligonucleotide primers used in PCR for dHPLC had been reported previously [1, 4, 12]. PCR products from analyzed samples were denatured and then cooled slowly to room temperature before dHPLC to maximize heteroduplex formation. The precise gradients for temperature and buffers giving the optimal TM for each fragment were calculated by WAVE-Maker 4.1 software (Transgenomic Inc., USA). The sequence variations could be detected using automated sequencing of variant fragments observed by dHPLC. Analysis of DNA sequencing was carried out on the ABI Prism 3100 Avant Genetic Analyzer (Applied Biosystems, Foster City, CA).

Methylation-specific PCR was used for methylation analysis of the APC promoter. Briefly, 1 *μ*g DNA was treated with sodium bisulfite using the CpGenome kit (Cat#S7820, Chemicon, USA) according to manufacturer's instructions. DNA methylation in the CpG islands of APC was determined by methylation-specific PCR. Primer sequences of APC promoter for the unmethylated DNA were 5′-GGGCTAGGGCTAGGCAGG -3′ (sense) and 5′-GCTGCACCAATACAGCCAC-3′ (antisense) amplifying the 98-bp product, while those for the methylated DNA were 5′-GGGTTAGGGTTAGGTAGG-3′ (sense) and 5′-AACTACACCAATACAACCAC-3′ (antisense). The PCR reaction was carried out using a hot start with a denaturation cycle of 94°C for 2 min and 80°C for 3 min, followed by 30 cycles of 30 s at 95°C, 30 s at 55°C, 40 s at 72°C, and final extension for 5 min at 72°C. The specificity of the reactions for methylated DNA is confirmed using unmethylated human sperm DNA and CpGenome Universal Methylated DNA.

## 3. Results

In this study, systematic screening strategy including MLPA, dHPLC, and sequencing was used to enable optimal mutation detection of germline and somatic mutations of *APC* gene in this FAP family.

### 3.1. Germline Mutations

**Figure 2:**
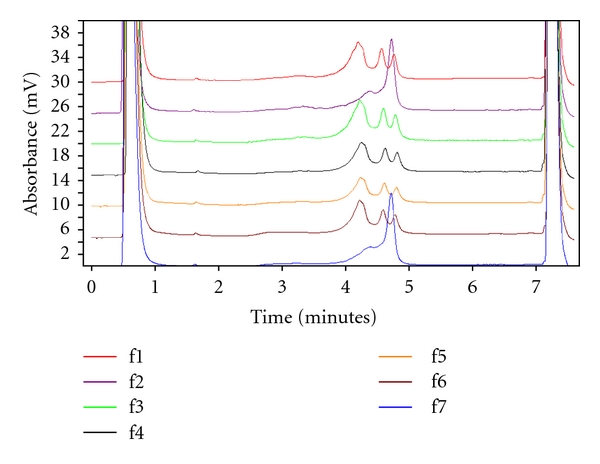
*APC* exon 15A by denaturing high-performance liquid chromatography (dHPLC) in the FAP pedigree family. f1 is the proband; f2 and f7 are the unaffected members; f3–f6 are the affected members.

**Figure 3:**
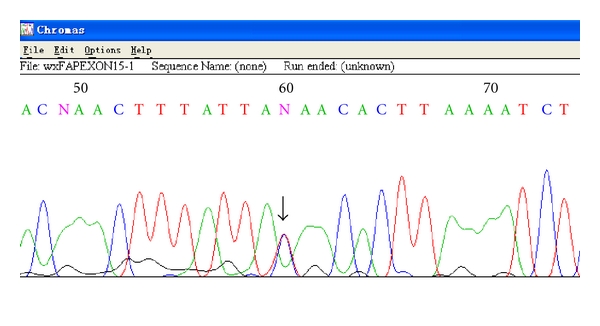
Sequence analysis: sequence spanning c.1999 C>T of individual 3, an affected family member. Arrow indicates a double peak at the site of the single nucleotide mutation, which leads to Q667X.

The whole coding region of *APC*, exon by exon, was subsequently screened for mutation by dHPLC in constitutional DNA from this family. Only one aberrant peak elution profiles in the fragment of exon 15A from constitutional DNA were shown for each of Number 1, 3, 4, 5, and 6 (Figure 2), which indicates they harbor the identical germline mutation. Sequence analysis revealed a c.1999 C>T substitution predicted to cause the substitution at codon 667 from Glutamine to STOP (Q667X) (Figure 3). While the 2 unaffected family members and 15 normal individuals as control were homozygous C at the same position. By MLPA, large deletion or duplication of *APC* gene was not found in constitutional DNA. All members and 15 controls showed consistently normal control peak pattern as described by the kit's manufacturer.

### 3.2. Somatic Mutations

**Figure 4:**
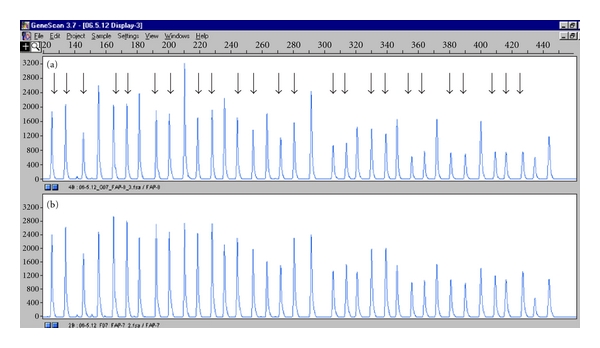
Peak pattern of exons from *APC* genes in MLPA. The *x*-axis shows the size of PCR products, and the *y*-axis reflects the relative quantity of PCR products. The arrows indicate a deletion of promotor region 1-2, exon 1–15 in the *APC* gene discovered in the adenomas from Number 3 of the family (a). The amount of PCR products in the *APC* gene was only half of a normal control (b).

**Figure 5:**
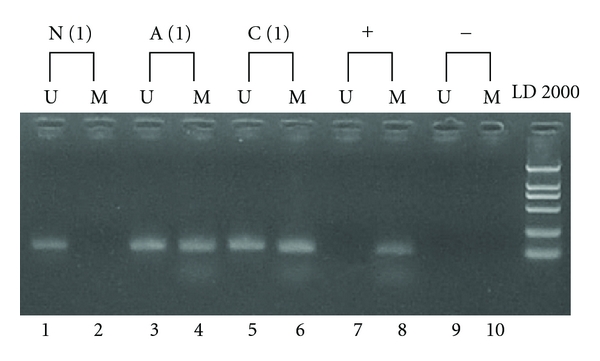
Methylation of the APC promoter was exclusively identified in the adenomas and carcinomas from Number 1. Primer sets for methylation-specific PCR are designated as methylated (M) and unmethylated (U) DNA. N (1): normal cancer-free tissue from Number 1. A (1): Adenoma from Number 1. C (1): Carcinoma from Number 1. +: Positive control. −: blank control.

Somatic mutations of *APC* gene were screening by dHPLC and MLPA in the collected tissues from this family. Out of 5 affected members' tissues, the adenomas from Number 3 were exclusively found somatic loss of wild-type *APC* allele (Figure 4). The APC promoter methylation was exclusively identified in both carcinomas and adenomas from Number 1 and in none of the other members (Figure 5).

### 3.3. Clinical Manifestation of the Family Members

Colonoscopic examination performed in the affected members (nos. 1, 3, 4, 5, and 6) found that the number of colonic polyps presented in the colorectum substantially increased along with their growth older. For Number 1 with additional APC promoter, hypermethylation has a severe form of FAP with colon carcinomas and hepatoblastoma, the typical extracolonic manifestations. For Number 3 with loss of wild-type allele, the severe phenotype was manifested with tubular adenomas with mainly heavy-grade dysplasia in colon and gastric fundal polyps. No polyps were found in the colonoscopy performed in Numbers 2 and 7, corresponding to their *APC*-negative mutation.

## 4. Discussion

Examination of *APC* germline mutations in FAP families is being used as an efficient tool for predictive testing of subjects at risk. However, its relevance for clinical management of individual patients is still open to question.

By dHPLC and sequence analysis, we found an *APC* Q667X in constitutional DNA, which was identical in 5 affected members and also presented in their tissues. The affected members have manifested an aggressive form of FAP with early onset of colonic adenoma and extracolonic manifestations, and the unaffected members have shown normal phenotype. We suggest the germline mutation of Q667X is the cause of clinical phenotype of the family with FAP.

Recent studies revealed that 5% of classic FAP was ascribed to the large deletions in the *APC*, which cannot easily be detected by conventional mutation-detection techniques [6, 13]. Therefore, we used MLPA test in our mutation analysis strategy to screen for exon-spanning deletions in this family though no large deletion was found in the constitutional DNA from this family. However, Number 3 was found somatic loss of wild-type *APC* allele in the adenomas.

The APC protein contains seven 20-amino acid repeats (20AARs) which are involved in degrading the transcriptional cofactor beta-catenin and hence negatively regulate Wnt signaling [14, 15]. The germline mutation Q667X encoded a limited amount of functional APC protein (23.4% of normal APC protein) and led to the truncated protein, which leaves zero 20AARs, implying a potential unfolding of beta-catenin. Moreover, the somatic loss of wild-type *APC* allele, as the “second hit”, was found in the adenomas from Number 3. Interestingly, the member carrying this somatic mutation had a more severe form of FAP with extracolonic manifestation though severe dysplasia may accumulate over time. We suggested that the somatic mutation may not only reduce the residual activity of the mutant germline allele but also inactivate the *APC* wild-type allele, and thus probably initiate the tumorigenesis. Several studies [16, 17] have shown that usually germline mutations between codons 1285 and 1378 were strongly associated with somatic loss of the wild-type *APC* allele in colorectal polyps in Caucasian population, which was inconsistent with our study. We thought it was the unique somatic *APC* mutation pattern that presents the clinical manifestation in this Chinese family pedigree.

The multitude of genetic changes may originate with a single mutation, which evolves towards tumorigenesis in conjunction with other genetic and epigenetic abnormalities, in particular CpG island hypermethylation [18]. In this family, we suggested that the promoter hypermethylation observed in adenomas and carcinomas from the proband may inactivate the second allele of APC and manifest the severe phenotype. These changes sometimes appear to be accumulated in all of the stages of colorectal tumorigenesis.

In FAP, correlations between site of mutation in the *APC* gene and severity of colonic polyposis or extracolonic manifestations are demonstrated [19–21]. However, several findings indicated that the geographic differences in APC mutation pattern may manifest population differences in the FAP phenotype [22–24]. Therefore, further studies with more Chinese family pedigrees analysis are necessary to give the overall view of the mutation pattern and to evaluate the genotype-phenotype association in Chinese FAP families compared to FAP families in other populations.
